# Improving Image Quality of Bronchial Arteries with Virtual Monochromatic Spectral CT Images

**DOI:** 10.1371/journal.pone.0150985

**Published:** 2016-03-11

**Authors:** Guangming Ma, Taiping He, Yong Yu, Haifeng Duan, Chuangbo Yang

**Affiliations:** Affiliated Hospital of Shannxi University of Traditional Chinese Medicine Radiology Dept, Xianyang City, Shaanxi, China; The First affiliated Hospital of Xi’an Jiaotong University, CHINA

## Abstract

**Objective:**

To evaluate the clinical value of using monochromatic images in spectral CT pulmonary angiography to improve image quality of bronchial arteries.

**Methods:**

We retrospectively analyzed the chest CT images of 38 patients who underwent contrast-enhanced spectral CT. These images included a set of 140kVp polychromatic images and the default 70keV monochromatic images. Using the standard Gemstone Spectral Imaging (GSI) viewer on an advanced workstation (AW4.6,GE Healthcare), an optimal energy level (in keV) for obtaining the best contrast-to-noise ratio (CNR) for the artery could be automatically obtained. The signal-to-noise ratio (SNR), CNR and objective image quality score (1–5) for these 3 image sets (140kVp, 70keV and optimal energy level) were obtained and, statistically compared. The image quality score consistency between the two observers was also evaluated using Kappa test.

**Results:**

The optimal energy levels for obtaining the best CNR were 62.58±2.74keV.SNR and CNR from the 140kVp polychromatic, 70keV and optimal keV monochromatic images were (16.44±5.85, 13.24±5.52), (20.79±7.45, 16.69±6.27) and (24.9±9.91, 20.53±8.46), respectively. The corresponding subjective image quality scores were 1.97±0.82, 3.24±0.75, and 4.47±0.60. SNR, CNR and subjective scores had significant difference among groups (all *p*<0.001). The optimal keV monochromatic images were superior to the 70keV monochromatic and 140kVp polychromatic images, and there was high agreement between the two observers on image quality score (kappa>0.80).

**Conclusions:**

Virtual monochromatic images at approximately 63keV in dual-energy spectral CT pulmonary angiography yielded the best CNR and highest diagnostic confidence for imaging bronchial arteries.

## Introduction

Dual energy spectral CT imaging has certain advantages in displaying vessels[1–4], which provides opportunity to improve the quality of spectral CT pulmonary angiography (CTPA) in imaging bronchial arteries. Since minimally invasive thoracoscopic esophageal and bronchial artery interventional surgical treatment for lung cancer is gradually increasing, it is significant for us to discern the bronchial artery before operation. However, no related report about the difference between the virtual monochromatic spectral CT images and the conventional polychromatic CT images in bronchial artery imaging is available at present. Therefore, the purpose of this study was to provide important evidence for using monochromatic images in spectral CT pulmonary angiography in imaging bronchial arteries at clinical application, by means of comparing the 140kVp polychromatic images, the default 70keV monochromatic images and the optimal keV monochromatic images.

## Material and Methods

### General Information

This retrospective study was approved by the Ethics Committee of Affiliated Hospital of the Shannxi University of Traditional Chinese Medicine, and patient consent was waived. The study group consisted of 38 patients (21 men and 17 women with mean age of 58 years) who underwent contrast-enhanced CTPA using spectral imaging mode for thoracic diseases between January and April, 2014 in our hospital. Exclusion criteria were: (1) patients with little subcutaneous fat, and (2) patients with severe image artifacts. All patients were confirmed by pathology after surgery or clinical certification. They consisted of 16 cases of lung cancer, 5 cases of esophageal cancer, 6 cases of pulmonary tuberculosis, 4 cases of chronic inflammation of the lungs, 4 cases of bronchiectasis, 1 case of mesothelioma and 2 cases of thymoma.

### Scan Technique

All scans were performed on a GE Discovery CT750HD (GE Healthcare, Waukesha, WI USA). Scanning range was from the level of the thoracic inlet to the diaphragm. The contrast-enhanced CTPA was acquired using the spectral CT imaging mode with the single tube, fast tube voltage switching between 80kVp and 140kVp. The Gemstone Spectral Imaging (GSI) protocol with tube current of 360mA and gantry rotation speed of 0.5s was selected (GSI-52). The other scan parameters were: 0.5s gantry rotational speed, 1.375:1 pitch, 5mm scan slice thickness, and 50cm scan field-of-view (SFOV). The volumetric CT dose index (CTDIvol) for the spectral CT imaging was 7.7mGy, similar to the 7.79±2.64mGy for the conventional single energy scans in our institution. A nonionic contrast agent (Iohexol, concentration 350mgI/ml) was injected at a dose of 1.0ml/kg, and flow rate of 4.0ml/s with a power injector through veins in the forearm. This was followed by 40ml saline injected at the same flow rate. The bolus tracking technique was used to determine the scan start time for the enhance scanning by placing a region of interest (ROI) on the bronchial bifurcation and the descending aorta level as a monitoring position. Enhanced CT scan was triggered with a threshold value of 150Hu.

### Image Generation and Data Analysis

101 sets of virtual monochromatic images with photon energy levels from 40keV to 140keV, and a set of polychromatic images corresponding to the conventional 140kVp tube voltage were reconstructed from the spectral CT scan for each patient with a reconstruction interval and reconstruction thickness of 0.625mm. These images were transferred to an advanced workstation (AW4.6, GE Healthcare) for image review and analysis with the Gemstone Spectral Imaging (GSI) Viewer software. Circular regions-of-interest (ROI) were placed in the center of bronchial artery and the size of the ROI were3~4mm^2^, on the peripheral interstitial of bronchial artery and the subcutaneous fat of the posterior chest wall to measure CT number and its standard deviation. Signal-to-noise ratio (SNR) and contrast-to-noise ratio (CNR) for bronchial artery were calculated using the following formula: SNR = CT_1_/SD, and CNR = (CT_1_-CT_2_)/SD, in which CT_1_ represents the CT attenuation value of the bronchial artery, CT_2_ refers to the CT attenuation value of the peripheral interstitial of bronchial artery within the same layer of mediastinum, and SD is the standard deviation measured on the subcutaneous fat. GSI Viewer automatically propagates the CNR calculation to all energy levels from 40keV to 140keV, and from which the optimal energy level for obtaining the highest CNR can be determined. Since the 70keV monochromatic image in spectral CT provides similar CT number in the muscle to the conventional 120kVp imaging[5], the SNR and CNR measurements from 70keV images were as the standard of reference for comparison with those of the optimal keV images and 140kVp polychromatic images.ROI placement was repeated three times for each image set to repeat CT_1_,CT_2_ and SD measurement three times. The final values were the average of the three measurements, The CT number and SD measurement were used to calculated the signal-to-noise ratio (SNR) and contrast-to-noise-ratio (CNR). The above three sets of axial images (at the optimal keV, 70keV and 140kVp) were further reformatted on AW into the volume rendering (VR) and maximum intensity projection (MIP) images for subjective image quality evaluation in terms of contrast enhancement in the vessel, vessel edge sharpness and vessel clarity using the 5-point scoring criteria listed in Table 1. For subjective assessment, two radiologists (H.F.D and Y.X.L, with 10 and 5 years of experience in chest CT, respectively) blinded to image types interpreted the images.

**Table 1:** Scoring criteria for bronchial artery.

scores	level	Description
**5**	excellent	Excellent opacification to the segmental level, very sharpedge
**4**	good	Good opacification to the segmental level, sharpedge
**3**	moderate	Limited opacification, somewhat blurred edge, diagnostic
**2**	poor	Suboptimal opacification, blurred edge, low confidence in making diagnosis
**1**	worse	Poor opacification, blurred edge, non-diagnostic

### Statistical Analysis

Quantitative measurements (SNR, CNR and subjective image quality score) were expressed as mean ±standard deviation (SD). Statistical analysis was carried out using SPSS17.0 soft ware to compare the three image sets of different energy levels (optimal keV, 70keV and 140kVp)[6]. The agreement of the two radiologists with regard to the image quality score was assessed using Cohen’s kappa test. The interval scales (SNR and CNR) were compared with one-way analysis. If there was statistically significant difference, LSD-test was carried out between any two groups. The objective image quality scores were compared with Kruskal–Wallis one-way ANOVA. If there was statistically significant difference, Wilcoxon signed rank test was carried out between any two groups. The p value of <0.05 was considered statistically significant.

## Results

### Objective Measurement

**Fig 1:**
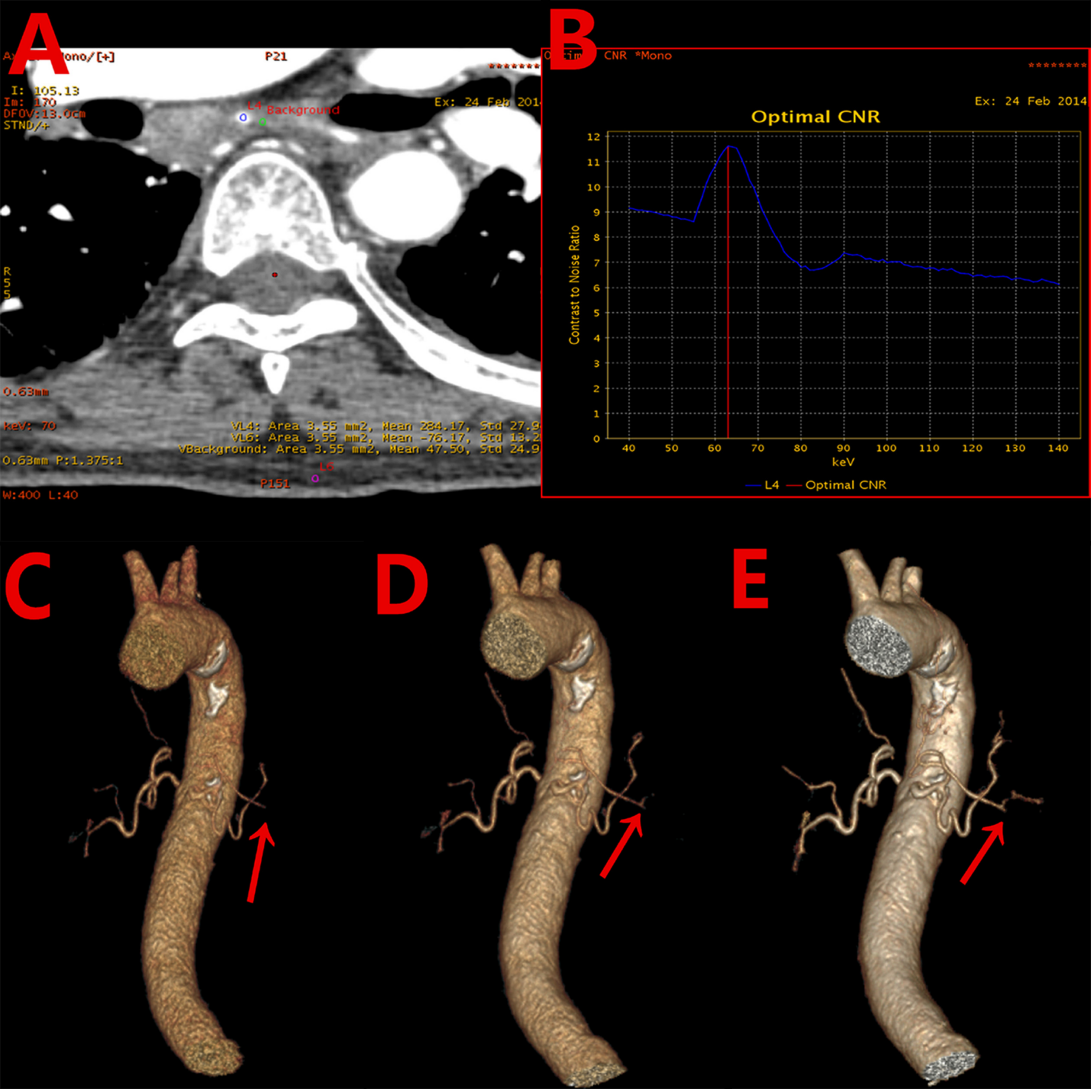
A 68 years old male patient with cancer in the right lung. (A) Axial image with region-of-interest indications for measuring CT number and standard deviation: bronchial artery blood vessels (blue), adjacent tissues in mediastinum (green), and subcutaneous fat on chest wall (purple). (B) Plot of contrast-to-noise ratio(CNR) as function of photon energy showing the optimal energy level of 63keV to obtain the highest CNR for the bronchial artery. (C) Volume-rendering (VR) 140kVp image with image quality score of 3. (D) VR image at 70 keV with image quality score of 4. (E) VR image at the optimal 63 keV with image quality score of 5.

The optimal energy levels to obtain the highest CNR for bronchial artery were 62.58 ± 2.74keV (54 keV—66 keV). Fig 1A shows an example of the generation of CNR for bronchial artery and (Fig 1B) shows an example of the CNR as function of photon energy. Fig 1B indicated that the optimal energy for achieving the highest CNR for this patient was at 63 keV. The CT numbers, SNR, CNR values for the three energy groups are listed in Table 2. The SNR and CNR values in the optimal keV monochromatic images increased by 18.85% and 18.83%, respectively, compared with the 70keV monochromatic images, and by 51.52% and 55.41%, respectively, compared with the 140kVp polychromatic images. The differences for SNR and CNR among the three energy groups were statistically significant.

**Table 2:** Comparison of signal-to-noise ratio (SNR), contrast-to-noise ratio (CNR) and Subjective image quality score for bronchial artery among images of three energy levels.

Group	N	CT_1_*	CT_2_**	SNR	CNR	Subjective image quality score
**optimal keV**^**a**^	38	275.14±69.31	48.67±36.53	24.9±9.91	20.53±8.46	4.47±0.60
**70keV**^**b**^	38	223.09±40.40	40.75±33.65	20.79±7.45	16.69±6.27	3.24±0.75
**140kVp**^**c**^	38	207.61±39.24	38.68±27.03	16.44±5.85	13.24±5.52	1.97±0.82
***P***				<0.001	<0.001	<0.001

* and ** represent the CT attenuation value in the bronchial artery and the peripheral interstitial of bronchial artery, respectively. The signal-to-noise ratio (SNR) and contrast-to-noise ratio (CNR) were compared with one-way analysis among the three groups and LSD-t test between two groups, p value all<0.05. The objective image quality scores were compared with Kruskal–Wallis one-way ANOVA among the three groups and Wilcoxon signed rank test between two groups, p value all<0.001. SNR: a vs b, *p*<0.05, a vs c, *p*<0.05 and b vs c, *p*<0.05; CNR: a vs b, *p*<0.05, a vs c, *p*<0.05 and b vs c, *p*<0.05; Subjective score: a vs b, *p*<0.001, a vs c, *p*<0.001 and b vs c, *p*<0.001.

### Subjective Image Quality Scores

The two radiologists had good agreement for subjective image quality score (Kappa>0.80). The scores from observer 1 for the three energy groups were analyzed. Subjective image quality scores are listed in Table 2 as well. The differences for the subjective image quality score among the three energy groups were statistically significant (x^2^ = 76.76, *p*<0.001). For the optimal keV monochromatic images, there were 20 cases with score of 5 (52.6%), 16 cases with score of 4 (42.1%), and 2 cases with score of 3 (5.3%); In comparison, there were 16 cases with score of 4 (42.1%), 15 cases with score of 3 (39.5%), and 7 cases with score of 2 (18.4%) for the 70keV monochromatic images; and for the 140kVp polychromatic images, there were only 1 case with score of 4 (2.6%), 9 cases with score of 3 (23.7%), 16 cases with score of 2 (42.1%), and 12 cases with score of 1 (31.6%), (Fig 1C–1E).

## Discussion

In this paper we studied the clinical applications of using spectral CT imaging to improve the depiction of bronchial arteries in CT pulmonary angiography. We have demonstrated that optimal energy level existed in spectral CT pulmonary angiography to provide the best contrast-to-noise ratio for displaying bronchial arteries. The clear depiction of bronchial artery is very important for determining the blood supplies to lung tumors[7] and the origin and extent of hemoptysis[8,9].Arterial infusion chemotherapy and bronchial arterial embolism are often used on hemoptysis, and the preoperative understanding of the 3D anatomy, such as the number of bronchial artery, its origin and course, as well as diameter, will contribute to the formulation and implementation of the treatment. For interventional treatment of lung cancer patients, it also can effectively reduce the suppository by mistake or omission of blood vessels, thereby reducing the complications of interventional treatment[10–12]. Currently, multi-slice CT (MDCT) bronchial arteriography is the main means of preoperative vascular evaluation, as it is non-invasive and has high spatial resolution. However, traditional MDCT with its polychromatic X-ray spectrum generates beam hardening artifacts that affect the image quality, and reduces low contrast resolution due to the average effect of X-ray photons with different energies in the spectrum. On the other hand, dual energy spectral CT imaging generates a set of virtual monochromatic images through the material decomposition process enabled by the use of the information from the two energy spectra of high and low kVp projections. The use of a monochromatic X-ray beam in CT would greatly reduce the averaging attenuation effect and increases contrast resolution[13]. The generation of virtual monochromatic image sets of various energy levels, especially low photon energy images in spectral CT imaging also allows us to select the optimal energy level to maximize the contrast-to-noise ratio for bronchial arteries in contrast-enhanced CT scans. This is because the main ingredient for the contrast medium in CT is iodine which has high atomic numbers which has much greater attenuation increase compared with surrounding tissues as photon energy decreases. This phenomenon enables greater attenuation separation between contrast-enhanced vessels and soft tissues at lower photon energies[14]. However, image noise changes as photon energy, and in general image noise is higher at the lower end of the energy spectrum due to the reduced number of photons. By calculating the contrast-to-noise ratio for bronchial arteries as function of photon energy, one can easily determine the optimal energy level for obtaining the highest CNR to display bronchial arteries, achieving balance between contrast in the vessel and image noise. Our study indicated that the optimal energy in spectral CT pulmonary angiography for our patient population was 62.58 ± 2.74keV (54keV–66keV). The subjective image quality in terms of vessel enhancement, sharpness and clarity at the optimal energy level was also judged to be better. On the other hand, the improved contrast-to-noise ratio with the optimal energy level in spectral CT may also be converted into either contrast dose or radiation dose reduction if the main focus in clinical applications to maintain the same CNR value as the conventional CT scans. To maintain the same CNR value, either contrast dose may be reduced taking advantage of the higher enhancement at lower photon energy or image noise requirement may be relaxed for radiation dose reduction.

Our study did have several limitations. First, we only had a small number of patients in this preliminary study. Second, this study was focused on image quality evaluation, and there was no separation of lung disease. The influence of lung diseases to bronchial artery is different, which could probably cause measurement deviation in SNR and CNR. Third, images were reconstructed with the traditional filtered back-projection algorithm. The effects of iterative reconstruction on image quality and the selection of the optimal energy level need to be further studied.

In conclusion, dual-energy spectral CT pulmonary angiography generates images with multiple energy levels from 40keV to 140keV, and virtual monochromatic images at approximately 63keV yield the best contrast-to-noise ratio and highest diagnostic confidence for imaging bronchial arteries.

## Supporting Information

S1 Dataset Data for SNR and CNR. (XLS)

S1 Table The two observers score for image quality. (DOC)
