# Supplementary material for: Improving Image Quality of Bronchial Arteries with Virtual Monochromatic Spectral CT Images
Source: PLoS One. 2016 Mar 11;11(3):e0150985. doi: 10.1371/journal.pone.0150985 (PMC4788436; doi:10.1371/journal.pone.0150985)
Supplement: S1 Table — (DOC) [file pone.0150985.s002.doc]

**The two observers score for image quality**

|  | Observer 1 | | | | | Observer 2 | | | | |  |
| --- | --- | --- | --- | --- | --- | --- | --- | --- | --- | --- | --- |
| Group | 1point | 2point | 3 point | 4 point | 5 point | 1point | 2point | 3 point | 4 point | 5 point | Kappa values |
| optimal keV | 0 | 0 | 2 | 16 | 20 | 0 | 0 | 4 | 12 | 22 | 0.811 |
| 70keV | 0 | 7 | 15 | 16 | 0 | 0 | 10 | 12 | 16 | 0 | 0.878 |
| 140kVp | 12 | 16 | 9 | 1 | 0 | 10 | 18 | 8 | 2 | 0 | 0.881 |

Note: The two observers score for image quality good consistency, Kappa values > 0.800.
